# Transition tips: Registering with the Health Professions Council of South Africa as a specialist family physician

**DOI:** 10.4102/safp.v64i1.5501

**Published:** 2022-04-14

**Authors:** Chantelle C. van der Bijl

**Affiliations:** 1 Department of Family Medicine, Faculty of Health Science, University of the Free State, Bloemfontein, South Africa

Congratulations, you have completed your registrar training! You feel elated until you realise you have to go through all the administration of registering with the Health Professions Council of South Africa (HPCSA) as a specialist. Despite what some of your colleagues might tell you, it is not a very tiresome process.

Here are the steps and practical tips to register as a family medicine specialist with the HPCSA as informed by my own experience:

- Firstly, ensure you have satisfied the following necessary criteria to register as a family physician:
  ■ You have passed the Colleges of Medicine of South Africa (CMSA)’s Fellowship of the College of Family Physicians Part A written examination.
  ■ You have completed your research and passed the CMSA’s Fellowship of the College of Family Physicians Part B.
  ■ You have completed your registrar training time.
  ■ You have satisfied all the criteria to obtain your Masters of Medicine (MMed) in Family Medicine from your University.
- You should get the following HPCSA forms completed:
  ■ Form 19 – Application for registration an additional qualification or category^1^. This form should be completed twice, once for your MMed (Family Medicine) and again for your Fellowship of the College of Family Physician (FCFP). (1) Complete Parts A and B. (2) Submit the proof of payment of the registration fee paid. The updated amount and banking details are on the HPCSA website. In 2021, the amount was R500.00 per additional qualification; a copy of your marriage certificate (should you wish to register in your married surname); your original degree/diploma certificate (a copy will only be accepted if certified by an attorney in his or her capacity as notary public and bearing the official stamp.) Copies certified by a Commissioner of Oaths will not be accepted. Alternatively, you can hand in Section C duly completed by the University or College; If you complete Section C, the original official date stamp of the institution should also be on the form.
  ■ Form 21 – Application for registration as specialist/sub-specialist^2^. (1) This form must be completed in detail and correctly. Information regarding experience must be provided in chronological order. (2) Attach your service record with your experience and posts held from internship onward and provide the exact post held and time spent in each post (beginning and end dates must be clearly indicated). You will be able to get this from your human resources department in the respective hospitals you have worked. (3) Proof of payment of the registration fee paid. The updated amount and banking details are on the HPCSA website. The amount was R6590.00 in 2021.
  ■ Form 57 – Certificate relating to training in specialities and sub-specialist^3^. (1) This form must be signed by the head of your academic department, medical superintendent of the teaching/satellite hospital/department/facility and the Dean (Faculty/School of Medicine/Health Sciences of University). (2) There should also be a university date stamp on the form. (3) If training was conducted in several teaching hospitals with different training post numbers, each teaching hospital should confirm training by completing a different form 57.
- Other documents not specified, but recommended to also attach to your application, include:
  ■ Certified copy of your identity document.
  ■ Your ethics committee approval letter for your research.
  ■ Proof of your CMSA Pass letter and breakdown of results or copy of your degree certificate.
  ■ Proof of your MMed Pass letter and breakdown of results or copy of your degree certificate.
- The HPCSA has an online service option to register your additional qualifications and as a specialist online. You upload electronic versions of your documents online. However, you will still need to send the original forms to the HPCSA office.
- Alternatively, you do not have to do the online application, but you can just send the original forms by registered mail or courier to:
  ■ The Registrar, PO Box 205, Pretoria 0001 or
  ■ 553 Madiba Street, Arcadia, Pretoria 0083.
  ■ You can also drop it off at their office at the same address mentioned here.

Some additional tips:

- Try to get your registration with the HPCSA done as soon as possible. If there are any delays, it will not hamper you from applying for a post.
- Be sure to save up some money for your registration; it is quite a hefty amount.
- Complete the forms legibly and in black ink.
- Make sure you do not make any mistakes on the forms. The HPCSA accepts no alterations on the documents.
- Be sure to know your HPCSA board-approved registrar post number, as you will need this to complete your forms. Your human resources department or university will be able to provide you with this.
- A courier is the best option for sending your documents to the HPCSA if you can’t hand it in yourself. It is probably more reliable, because you will be able to track it.
- For further information, consult the HPCSA guideline for registration as a specialist^4^ on the HPCSA website.

The Next 5 is an official South African Academy of Family Physicians (SAAFP) initiative aimed at assisting newly qualified family physicians within their first 5 years of qualifying. ‘Transition tips’ is a regular feature in the *South African Family Practice* (SAFP) journal written with the Next 5 audience in mind and informed by a membership survey.^5^ If you are keen to learn more about this initiative and wish to become involved, please email to admin@saafp.org.
